# CircSLNN: Identifying RBP-Binding Sites on circRNAs *via* Sequence Labeling Neural Networks

**DOI:** 10.3389/fgene.2019.01184

**Published:** 2019-11-22

**Authors:** Yuqi Ju, Liangliang Yuan, Yang Yang, Hai Zhao

**Affiliations:** ^1^Center for Brain-Like Computing and Machine Intelligence, Department of Computer Science and Engineering, Shanghai Jiao Tong University, Shanghai, China; ^2^Key Laboratory of Shanghai Education Commission for Intelligent Interaction and Cognitive Engineering, Shanghai Jiao Tong University, Shanghai, China; ^3^Brain Science and Technology Research Center, Shanghai Jiao Tong University, Shanghai, China

**Keywords:** RNA–protein binding sites, sequence labeling, convolutional neural network, bidirectional LSTM neural network, deep learning

## Abstract

The interactions between RNAs and RNA binding proteins (RBPs) are crucial for understanding post-transcriptional regulation mechanisms. A lot of computational tools have been developed to automatically predict the binding relationship between RNAs and RBPs. However, most of the methods can only predict the presence or absence of binding sites for a sequence fragment, without providing specific information on the position or length of the binding sites. Besides, the existing tools focus on the interaction between RBPs and linear RNAs, while the binding sites on circular RNAs (circRNAs) have been rarely studied. In this study, we model the prediction of binding sites on RNAs as a sequence labeling problem, and propose a new model called circSLNN to identify the specific location of RBP-binding sites on circRNAs. CircSLNN is driven by pretrained RNA embedding vectors and a composite labeling model. On our constructed circRNA datasets, our model has an average *F*_1_ score of 0.790. We assess the performance on full-length RNA sequences, the proposed model outperforms previous classification-based models by a large margin.

## Introduction

Benefitting from the rapid development of high-throughput experimental technologies, transcriptome, proteome, epigenome and other omics data have accumulated in an unprecedented speed. The multi-omics data have enabled large-scale studies on gene regulation at different levels. Especially, the interactions between RNAs and RNA binding proteins (RBPs) are crucial for understanding post-transcriptional regulation mechanisms (Filipowicz et al., 2008). The RNA–RBP-interactions play important roles in protein synthesis, gene fusion, alternative mRNA processing, etc. (Bolognani and Perrone-Bizzozero, 2008). The aberrant expression of RBPs and disruption of RNA–RBP-interactions are closely related to various diseases of human beings (Khalil and Rinn, 2011). In the early stage of RNA–RBP-interaction studies, the recognition of binding sites mainly relies on the analysis of RNA–protein complexes *via* biophysical methods. As the experimental process is costly and laborious, it is increasingly important to develop automatic tools to predict binding sites.

As for protein–protein-interactions, both structures and amino acid sequences are commonly used for identifying binding sites, including POCKET (Liu and Hu, 2011), Fpocket (Le Guilloux et al., 2009) LIGSITE (Hendlich et al., 1997), etc. The structural feature-based prediction methods exploit protein 3D structures and appropriate geometries to locate potential binding regions. Most structure-based methods assume that proteins bound to the same ligand have similar overall structure and biochemistry characteristics, while some researchers found that proteins having the same binding site may have diverse sequences or structures (Muppirala et al., 2011). Sequence-based methods usually utilize amino acid composition, function domain, secondary structure and solvent accessibility information (Shen et al., 2007).

Due to the lack of solved structures for RNA-protein complexes, most of the existing studies have turned to sequence information and machine learning methods for predicting RBP-binding sites on RNAs, like support vector machines (SVMs) (Kumar et al., 2008) and random forest (RF) (Liu et al., 2010). Moreover, deep learning models have emerged in this field (Alipanahi et al., 2015; Pan and Shen, 2017). Deep learning is a data-driven approach that allows automatic learning of the advanced features from data without the need for domain knowledge, by stacking multiple layers of neural networks (LeCun et al., 2015). Compared to traditional machine learning models, it does not require feature engineering and can achieve better performance. A few deep learning methods, including convolutional neural network (CNN) and recurrent neural network (RNN), have been developed to predict RBP-binding sites (Pan and Shen, 2017; Pan et al., 2018).

Although researchers have made some progress in predicting RNA–protein binding sites, current mainstream prediction methods have some limitations.

First, most prediction methods simplify the prediction task as a binary classification problem, i.e. they assign a positive/negative label to a segment of RNA, where the positive label denotes the presence of a binding site. Actually, binding sites on RNAs are sequence fragments that range from tens to hundreds of nucleotides in length. Thus, the prediction based on fixed-length fragments may be inaccurate, as it only yields approximate locations of binding sites and could not specify the length that the sites span.

Second, most of the existing methods predict the interaction between linear RNAs and RBPs, while circular RNAs (circRNAs) have been rarely studied. CircRNAs play an important role in gene regulation, and they also play crucial roles in the development of many complex diseases (Fan et al., 2018). Thanks to the advances of new sequencing technology, circRNAs have been identified on the whole genome scale (Song et al., 2016). Moreover, the interplay between circRNAs and proteins or microRNAs has attracted more and more research interests from biomedical field, resulting in large-scale data of circRNA–RBP interactions using high-throughput experiments, like CLIP-Seq (Dudekula et al., 2016). Thus, the models for predicting binding sites on circRNAs are in great demand.

In this study, we propose a sequence labeling neural network model to predict circRNA–protein binding sites, called circSLNN, which is composed of a long-short-term memory (LSTM) network, a convolutional neural network (CNN) and a conditional random field (CRF) model. Instead of performing a binary classification on the whole fragment, it assigns a label (bound or unbound) to each position on the fragment. Compared with traditional classifiers, it can not only predict whether the input segment is bound to a given RBP, but also predict the specific location of binding sites on the segment. Besides, in order to fully utilize the sequence information of circRNAs, we propose to use RNA embeddings learned *via* a similar word embedding algorithm for processing natural languages, where the corpus is extracted from the whole human genome. To the best of our knowledge, this is the first predictor for RNA–protein binding sites using a sequence labeling scheme. The contributions of this study are listed in the following.

1. We construct the sequence labeling network of LSTM-CNN-CRF for predicting RBP-binding sites on RNA sequences. Compared to previous methods, it has the advantage in identifying location and length of binding sites.
2. We apply RNA embeddings to the prediction of RNA–RBP interaction, and demonstrate the effectiveness of continuous dense feature vectors trained by word embedding and whole-genome corpus.
3. We propose a predictor, circSLNN, trained on circRNA binding sites, which may help researchers reveal the interaction mechanisms of circRNAs and proteins.

## Related Work

### Prediction Based on Traditional Machine Learning Methods

The prediction of molecular interactions has been a hot topic in bioinformatics over the past decades. Especially, the protein–protein-interactions (PPIs) have been well-studied due to the abundant information that can be utilized in the prediction, e.g. amino acid sequences, function domains, gene ontology annotation (Ashburner et al., 2000). The machine learning-based predictors usually consist of two parts, i.e. the feature extraction and classification. Similar to PPI, the prediction of RNA–RBP-interaction is a typical machine learning problem. However, due to the lack of functional annotation of RNAs, the feature extraction mainly relies on RNA sequences or secondary structures. For some types of RNAs, like circRNAs which have constrained structures, i.e. covalently closed continuous loops, the effective feature extraction from sequences are more important.

Traditional feature representation of RNA sequences include *k*-tuple composition, pseudo *k*-tuple composition (PseKNC) (Chen et al., 2013), etc. The features are discrete vectors, working with shallow learning models. For instance, Muppirala et al. (2011) used the SVMs and random forest methods to predict the RNA–RBP-interactions. As the rise of deep learning, sequence encoding schemes and deep neural networks have been emerging and achieved better prediction performance.

### Prediction Based on Deep Neural Networks

DeepBind (Alipanahi et al., 2015) is a pioneer work in developing deep learning models for RNA–RBP-interactions. The model is based on a convolutional neural network, which not only improves prediction accuracy but also reveals new sequence patterns at the binding area. Later, Pan et al. released a series of computational tools, including iDeep (Pan and Shen, 2017), iDeepS (Pan et al., 2018) and iDeepE (Pan and Shen, 2018), which have different feature representation and model architecture. iDeep utilizes five different information sources, i.e. secondary structure information, motif information for describing the conserved region of sequences, CLIP co-binding, region type, and sequence information, to extract high-level abstraction features *via* deep learning models. Especially, the sequence information is processed by a CNN (Krizhevsky et al., 2012), while other four data sources are processed by deep belief networks (Zou and Conzen, 2004). Compared with iDeep, iDeepS reduces the types of data sources and only retains sequence information and secondary structure information. The authors added bi-directional long short-term memory (BiLSTM) (Schuster and Paliwal, 1997) to integrate the data, which better reserves contextual information based on relative position relationship of nucleotides.

Generally, the performance of deep learning-based methods depends on informative feature representation and powerful model architecture. In this study, we explore both the two parts to improve prediction accuracy.

## Materials and Methods

### Data Source

To construct a predictor for circRNA–RBP-interactions, we collect a standard dataset of RBP-binding sites on circular RNAs from the circRNA Interactome database (Dudekula et al., 2016), which contains sequence information for more than 100,000 human circRNAs, as well as specific locations of binding sites for different RBPs. Each binding site is represented as an interval from the start index to the end index on the circRNAs. We extend 50-nt upstream and downstream respectively by taking the midpoint of each interval as the center. In this way, 101-nt fragments can be obtained as positive samples. Then we randomly extract 101-nt segments from the remaining fragments as negative samples. In order to avoid the issue caused by repeated sequences, we remove redundant sequences using CD-HIT (Li and Godzik, 2006). The positive-to-negative ratio is 1:1, and the training-to-test ratio is 5:1.

Then we generate standard labels for all samples. For positive samples, we label all the symbols within the binding sites as “I” and all the other locations as “O”, meanwhile we mark all symbols as “O” for negative samples. Here we use the IO tag scheme, where “I” is short for inside (a binding site) and “O” is short for outside, i.e. not a binding site. As it is known that, the BIO format (short for inside, outside, beginning) is a common tagging format in natural language. As there are a lot of adjacent labeling objects in text, it is hard to distinguish between different labeling objects using only the IO scheme. By contrast, in the sequence labeling problem of binding sites, the distribution of binding sites is extremely sparse, and usually binding segments are far from each other. Thus, we use the IO labeling scheme to reduce the types of labels and make the training model easier to converge.

### Data Encoding

As mentioned in the *Related Work* section, feature representation can have a substantial impact on the performance for both shallow learning and deep learning models. To work with deep models, RNA sequences need to be encoded into numerical vectors, like one-hot vectors. In recent years, more and more studies on biological sequence analysis have adopted word embedding-based encoding schemes to replace one-hot encoding (Harris and Harris, 2010), as embedding vectors are continuous and high-dimensional, which may capture more context and semantic information in sequences. In our previous studies, we propose the RNA2Vec method to get RNA embeddings (Xiao et al., 2018). We regard 10-mer segments as words and train the word embeddings using Glove (Pennington et al., 2014).

### Model Architecture

**Figure 1:**
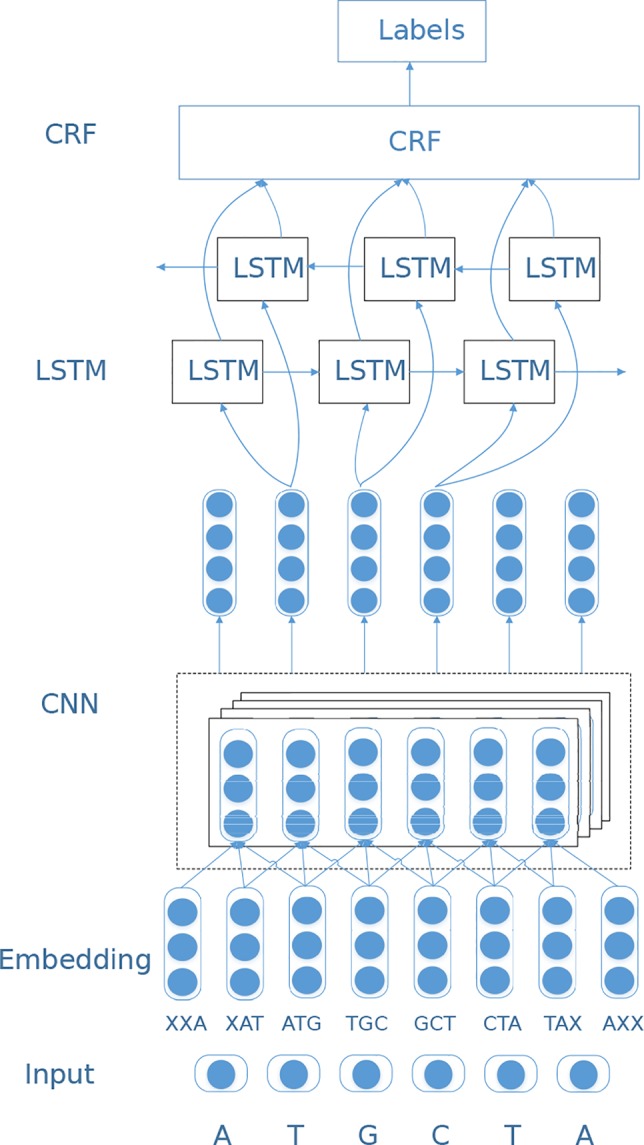
The overall architecture of CircSLNN.

In this study, we design a sequence labeling model based on deep neural networks to predict RBP-binding sites on RNAs. We first feed the embedding vectors to a convolutional neural network (Krizhevsky et al., 2012) to extract local features, and then learn the long-distance dependency information among bases through a BiLSTM layer. Finally, the label identification of the entire RNA sequence is completed by the CRF layer (Lafferty et al., 2001). The network structure is shown in **Figure 1**.

### CNN Layer

Convolutional neural network (CNN) (Krizhevsky et al., 2012) is a widely used deep learning architecture. CNN generates feature maps at different abstract levels by stacking convolutional layers. In circSLNN, the CNN serves as a feature extractor from the initial input vectors. As sequence labeling models predict a label for each symbol in the sequence, whereas the embedding vectors are trained for 10-mers, we adopt CNN to extract high-level features for each nucleotide in RNA sequences based on the embedding vectors of its surrounding 10-mers, i.e. a window centered by the nucleotide.

Specifically, for each individual nucleotide (except for the first 9 nucleotides), there are 10 fragments of length 10 containing it. Based on the vectors of the 10 fragments, we perform feature extraction *via* a one-dimensional CNN. Suppose the dimensionality of embedding vectors is *m*, then each nucleotide can be represented as a matrix of size 10×*m*, which is fed to the CNN. Before using CNN, we need to expand the 101-nt fragments to 110-nt (101 + 10 − 1), which is passed through a sliding window of size 10. Here we pad the matrix by zero vectors.

Let *h* *_j_* be the size of the *j*th convolutional kernel, *X* *_i_* be the matrix of the sliding window at the *i*th time step, which consists of the *i*th to the (*i* + *h* *_j_* − 1)th columns of the original input. Thus, the features learned by the convolutional layer can be expressed in Eq. 1,

(1) $$\begin{array}{r} c_{ij}=f(w_{j}*X_{i:i+hj-1}+b_{j})\\ i\in \{1,2,\ldots ,N-h_{j}+1\},\;j\in \{1,2,\ldots n\} \end{array}$$

where *n* is the number of filters, *f*(.) is the activation function, and *w* *_j_* and *b* *_j_* are the weight matrix and the offset, respectively.

### BiLSTM Layer

Till now, the mechanism of RNA–RBP-interaction has not been fully understood yet, and various factors impact the binding between RNAs and RBPs, include not only the local structural motifs and binding domains but also long-term dependencies of nucleotides. In our model, the CNN component serves as a feature extractor from raw input and learn the context information in local regions. To further exploit sequence information, we adopt bi-directional long short-term memory (BiLSTM) (Schuster and Paliwal, 1997) network. BiLSTM is a combination of forward LSTM and backward LSTM, which is a special type of recurrent neural network (RNN). It is often used to model context information in natural language processing tasks. BiLSTM was designed to learn the relationship between base before and after the current position, and to capture longer distance dependencies.

Let *x* *_t_* be the input vector of the *t*th time step, and *s* *_t_* and *s*ʹ*_t_* be the hidden states of the forward and backward calculations of the *t*th time step. Then the calculations of *s* *_t_* and *s*ʹ*_t_* depend on *s* *_t_*_-1_ and *s*ʹ*_t_*_+1_, respectively, as shown in Eqs. 2 and 3.

(2) $$s_{t}=g(Ux_{t}+Ws_{t-1})$$

(3) $${s^{\prime }}_{t}=g(U^{\prime }x_{t}+W^{\prime }{s^{\prime }}_{t+1})$$

where *U* and *W* are the weight matrices of the input and hidden states in the forward pass. *U*′ and *W*′ are the weight matrices of the input and hidden states in the backward pass.

The final output *o* *_t_* of step *t* is a combination of a forward hidden layer and a backward hidden layer, defined as follows.

(4) $$o_{t}=h(Vs_{t}+V^{\prime }{s^{\prime }}_{t})$$

where *V* and *V*′ are the weight matrices of the hidden layers to the output layer in forward pass and backward pass, respectively.

### CRF Layer

As mentioned in the *CNN Layer* and *BiLSTM Layer* sections, CNN and RNN have their respective advantages. The hybrid CNN-RNN architecture has been proposed in previous studies and achieved much better performance than using CNN or RNN alone. For instance, both CRIP (Zhang et al., 2018) and iDeepS (Pan et al., 2018) are hybrid CNN-RNN models, and both use LSTM for classification. CRIP feeds the outputs for all time-steps of the LSTM to a fully-connected layer and get the decision result, while iDeepS uses the output of the last time-step for classification. Actually, based on the output on each time-step of LSTM, it is straightforward to get the sequence labeling results. However, the raw outputs without any constraint are often meaningless, e.g. OIOI … OOI, as it is known that binding sites are continuous regions on RNA sequences. In order to avoid such cases, we add a conditional random field (CRF) layer to process the output of BiLSTM. The purpose of the CRF layer is to predict the probability of the entire sequence rather than the probability of each individual tag. The CRF layer can add some constraints to the predicted labels to ensure that the output labels are legal. During the data training process, these constraints can be automatically learned through the CRF layer, so the probability of occurrence of illegal sequences in the prediction phase will be greatly reduced. Specifically, the CRF layer calculates the conditional probability shown in Eq. 5

(5) $$P(y_{1},\ldots ,y_{n}|x_{1},\ldots ,x_{n})=P(y_{1},\ldots ,y_{n}|x),\;x=(x_{1},\ldots ,x_{n})$$

where *P*(*y*|*x*) is the probability that the prediction label is *y* if the input is *x*, where *x* *_i_* is the output of *i*th time-step by the LSTM layer.

In order to estimate the probability, CRF makes two assumptions. First, the distribution is an exponential family distribution. Second, the association between the outputs occurs only at adjacent locations, and the association is exponentially additive. This allows the probability to be calculated by the probability density function as shown in Eq. 6.

(6) $$\begin{array}{c} f(y_{1},\ldots ,y_{n};\;x)=h(y_{1};x)+g(y_{1,}\;y_{2};x)+h(y_{2};x)+\\ g(y2,\;y3;x)+h(y3;x)+\cdots +g(y_{n-1},\;y_{n};x)+h(y_{n};x) \end{array}$$

where *f*, *g*, *h* are probability density functions and can be considered as scoring functions. The overall score *f* of all tags can be broken down into the sum of the score *h* of each individual tag and the score *g* of each pair of adjacent tags. Since LSTM is capable to learn the mapping from input *x* and its output *y*, we assume that the function *g* is independent of *x* and the final probability distribution can be formulated in Eq. 7,

(7) $$P(y_{1},\ldots ,y_{n}|x)=\frac{1}{Z(x)}exp(h(y_{1};x)+\sum_{k=1}^{n-1}[g(y_{k},y_{k+1})+h(y_{k+1};x)])$$

where the single-label scoring function *h*(*y* *_i_*; *x*) is fitted by the BiLSTM layer, thus completing the construction of the CRF layer.

## Experimental Results

### Experimental Settings

In circSLNN, the number of convolution kernels in the CNN layer is 128, the convolution window size is 10, the hidden layer size of the BiLSTM layer is 256, and the activation function used by the middle layer is ReLU. The optimization algorithm is RMSProp, with batch size 512 and epoch number 20, using the early stopping mode. The performance metrics include precision, recall and *F*_1_, which are computed based on the labels of individual nucleotides.

### Prediction Performance of circSLNN

We perform experiments on all 37 datasets described in the **Data Source **section. For each dataset, we perform a 6-fold cross-validation. The original datasets are divided into 6 folds with approximately equal size (5 folds for training and validation, and one fold for test). The accuracies shown in **Table 1** are averaged over 6 times of independant test.

**Table 1:** Prediction accuracies on 37 different protein datasets.

Protein	Precision	Recall	*F*_1_-Measure
AGO1	0.820	0.853	0.836
AGO2	0.804	0.429	0.559
AGO3	0.840	0.773	0.805
ALKBH5	0.908	0.928	0.918
AUF1	0.908	0.938	0.923
C17ORF85	0.889	0.926	0.907
C22ORF28	0.847	0.828	0.838
CAPRIN1	0.881	0.789	0.833
DGCR8	0.794	0.863	0.827
EIF4A3	0.520	0.749	0.614
EWSR1	0.892	0.912	0.902
FMRP	0.473	0.679	0.557
FOX2	0.999	0.925	0.961
FUS	0.583	0.566	0.575
FXR1	0.958	0.951	0.955
FXR2	0.799	0.825	0.812
HNRNPC	0.841	0.892	0.866
HUR	0.542	0.609	0.573
IGF2BP1	0.522	0.716	0.604
IGF2BP2	0.691	0.660	0.675
IGF2BP3	0.533	0.618	0.572
LIN28A	0.543	0.702	0.613
LIN28B	0.764	0.636	0.694
METTL3	0.774	0.806	0.790
MOV10	0.805	0.808	0.806
PTB	0.609	0.597	0.603
PUM2	0.910	0.988	0.948
QKI	0.982	0.971	0.976
SFRS1	0.797	0.704	0.748
TAF15	0.916	0.968	0.941
TDP43	0.864	0.760	0.809
TIA1	0.915	0.863	0.888
TIAL1	0.836	0.824	0.829
TNRC6	0.952	0.841	0.893
U2AF65	0.848	0.796	0.821
WTAP	0.976	0.953	0.964
ZC3H78	0.848	0.790	0.818
Average	0.794	0.795	0.790

As can be seen, circSLNN achieves high prediction accuracy for most RBPs. The *F*_1_ scores are higher than 0.8 on 24 out of the 37 datasets, showing the effectiveness of the sequence labeling model.

### Data Encoding Analysis

**Figure 2:**
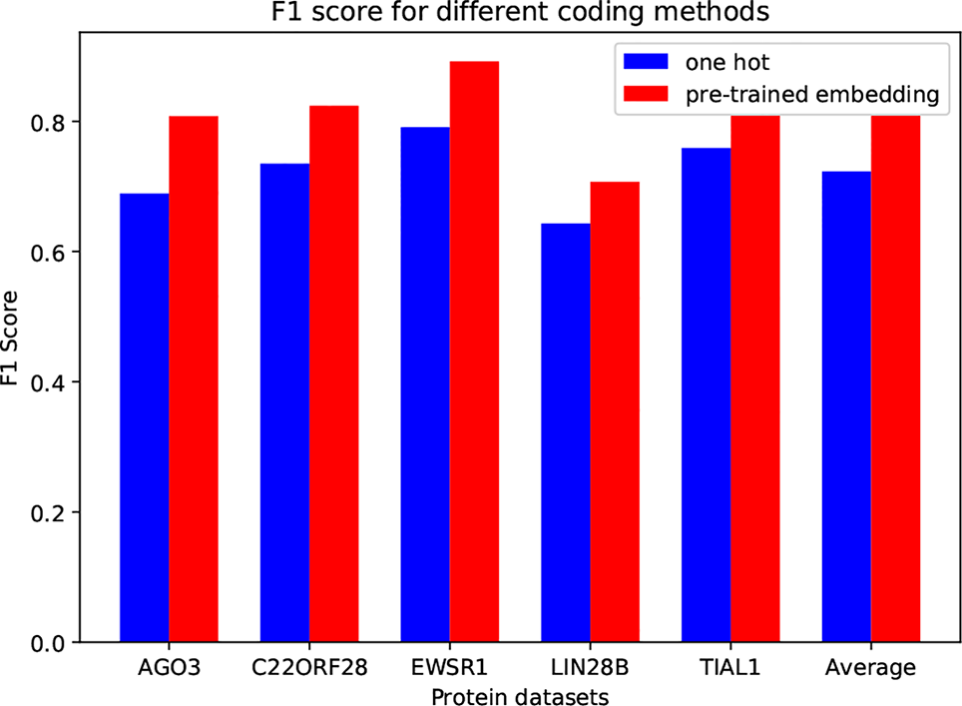
*F*_1_ Score for Different Coding Methods.

In circSLNN, the inputs are pretrained embedding vectors for *k*-mers, while most of the existing methods for predicting RBP-binding sites use one-hot encoding, e.g. iDeep and DeepBind. In order to investigate the impact of encoding scheme on model performance, we compare one-hot and our embedding vectors on the same datasets. We randomly choose 5 RBPs. **Figure 2** depicts the comparison results.

Apparently, the pretrained embedding vectors perform much better than the one-hot vectors. The average *F* *_1_* score is increased by 0.087. This result suggests that the word embedding encoding method can effectively extract the feature information of RNA sequences from the human genome database, and can effectively improve the performance of the binding site predictor.

### The Role of CNN Layer

**Figure 3:**
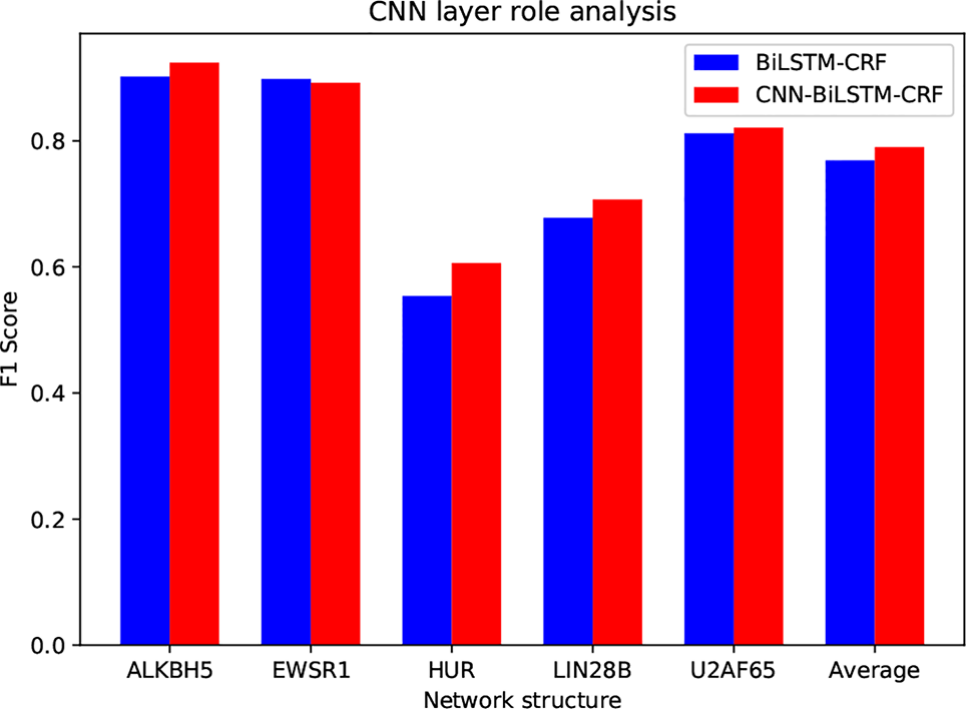
Performance comparison between models with and without the CNN layer.

Compared to ordinary text sequence labeling tasks, we introduce the CNN layer to extract local features from RNA sequences. The purpose of the CNN layer is to characterize the local sequence pattern surrounding the base to be labeled, and encode each individual base with richer information. Here we assess the contribution of CNN by removing it from the model. The inputs of the LSTM-CRF model are the pretrained *k*-mer embedding vectors. Specifically, for each base, we choose the embedding vector of the fragment that centered by the base as its feature vector. The following training on LSTM and CRF is the same as circSLNN. We compare the performance of the two methods on five randomly selected data sets, as shown in **Figure 3**.

As can be seen, the average *F*_1_ is increased by 0.021 by introducing CNN layer. Although the overall improvement seems not significant, we find that CNN has larger contribution for the difficult datasets, e.g. HUR and LIN288, compared with easy datasets, indicating the importance of further feature learning from raw inputs.

### Comparison of Different Sequence Labeling Schemes

**Figure 4:**
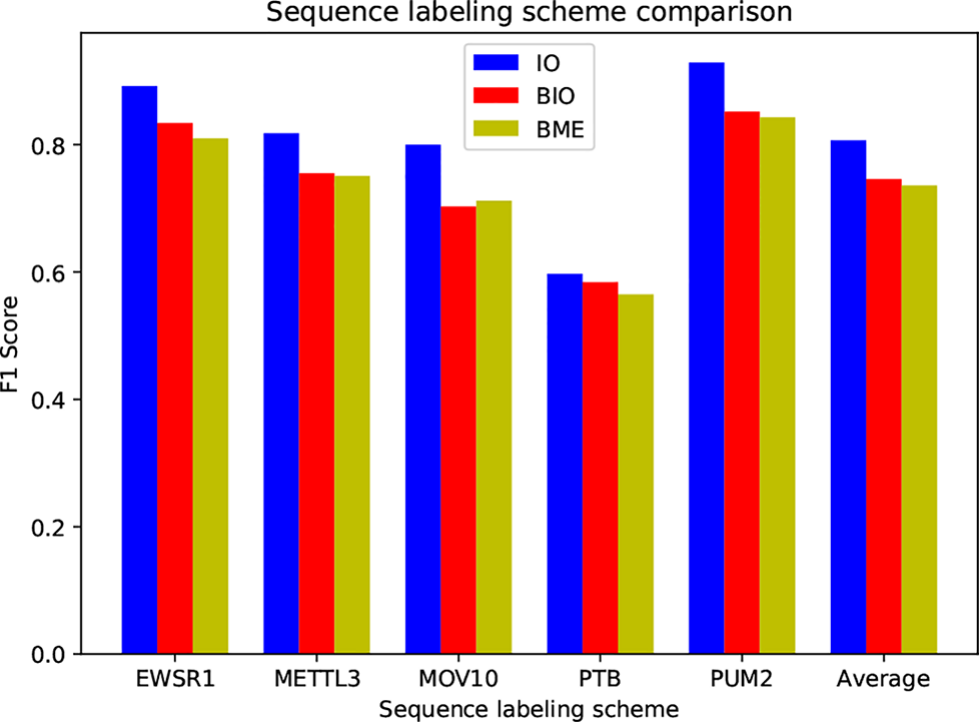
Performance comparison on three sequence labeling schemes.

The sequence labeling scheme used in this study is IO tag, not the BIO or BME (BME is short for begin, middle and end) that commonly used in text labeling tasks (Carpenter, 2009), as binding sites generally span tens of bases in length, whereas common text labeling objects only consist of several words, such as a typical place name in the named entity recognition mission (NER), ‘Shanghai Jiao Tong University’. In order to assess the performance of these three tag systems, we conduct experiments on five randomly selected protein datasets, as shown in **Figure 4**.

As can be seen, the IO tag system outperforms BIO and BME by a large margin. BIO and BME have close performance. We find that the B-coded labeling systems can hardly find tag B in the test set, i.e. their results contain only tag I and tag O. The reason is that the B tag is extremely sparse due to the long binding sites, which leads to an imbalanced distribution of tags, and it is very hard to recognize tag B.

### Investigation on Positive-to-Negative Data Ratio

**Figure 5:**
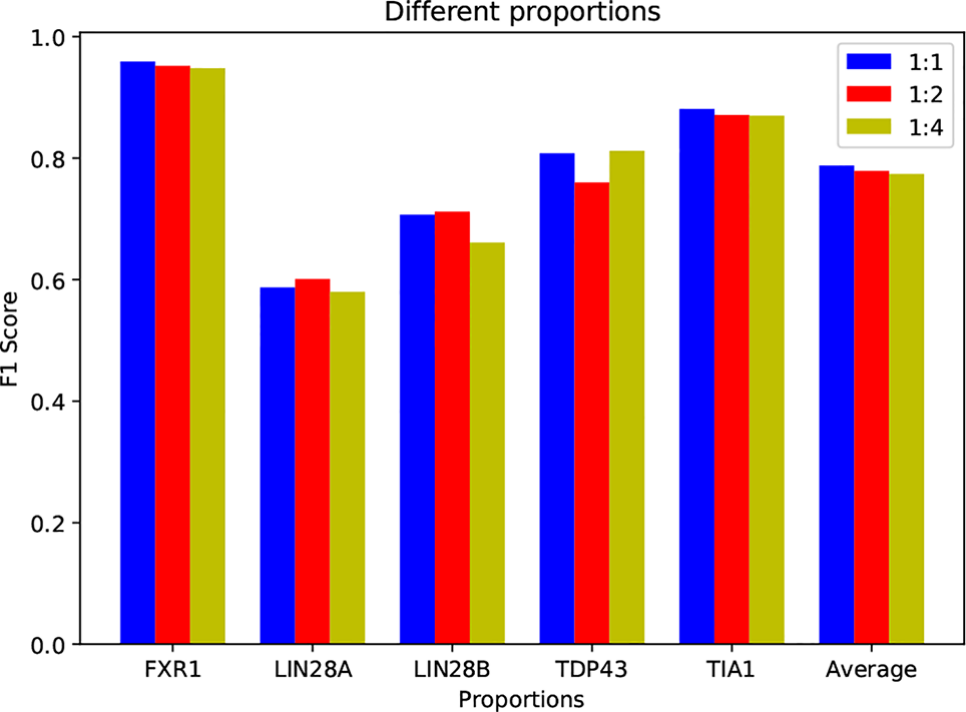
Performance on datasets with different positive-to-negative data ratios.

In our experiments, the positive-to-negative ratio for all datasets is 1:1, which is the same as previous studies (Pan and Shen, 2017), (Zhang et al., 2018). However, the length of human circRNAs could be tens of thousands bases, including 1–5 exons (Memczak et al., 2013), while the binding sites are small regions and very sparse on the sequences. That is to say, the true ratio between positive and negative data is very small, leading to an extremely imbalanced problem, thus most studies adopt a sampling strategy to control the ratio. Here, to get closer to the actual situation, we compare the performance of circSLNN under different positive-to-negative ratios, i.e. 1:1, 1:2, and 1:4. The results are shown in **Figure 5**.

Note that although adding negative samples results into data imbalance, the increase in data volume is beneficial for training the model. As shown in **Figure 5**, the accuracies on some datasets, e.g. LIN28B, LIN28B, and TDP43, have even been increased by using expanded negative set. Generally, the performance of circSLNN has little variance when expanding negative set several times, showing the model robustness.

### Comparison With the Existing Methods on Sequence Labeling for Full-Length circRNAS

In order to assess the performance of circSLNN in real cases, we conduct experiments on full-length circRNAs instead of sampled segments in the datasets, and compare it with the state-of-the-art predictors for RNA–RBP binding sites.

To the best of our knowledge, circSLNN is the first sequence labeling model for identifying RBP-binding sites on circRNAs. Therefore, for the convenience of comparison, we need to process the output of the existing classification models, i.e. converting the labels for segments into labels for individual nucleotides. Specifically, for a full-length RNA, we divide it from beginning to end into 101-nt fragments. For each fragment, the circSLNN model is used to predict whether each base belongs to the binding site. If it belongs, it is marked as 1; otherwise, it is marked as 0. For the classification model, whether the fragment belongs to the binding site is predicted. If the fragment is predicted as positive, then all the bases in the sequence are labeled by 1, otherwise all bases are labeled by 0. In this way, we obtain the label sequences of full-length RNAs predicted by two different models. By comparing the predicted sequence labels with the actual labels, we can calculate the *F*_1_ score.

**Figure 6:**
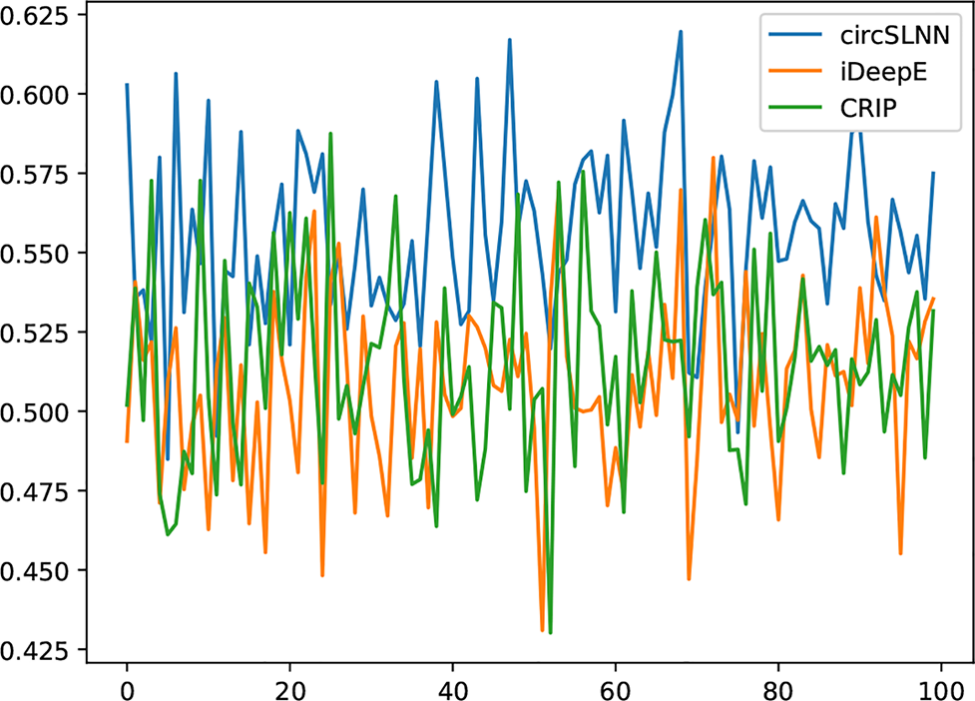
*F*_1_ score on 100 full-length RNAs.

We collect a dataset of 100 full-length circRNAs that are bound to different RBPs. They are first segmented into 101-nt segments, and then fed to the classification models and sequence labeling model, respectively, to predict the binding sites. *F*_1_ scores are computed based on individual bases. The results are shown in **Figure 6**.

As can be seen from the results, circSLNN achieves the highest *F*_1_ on almost all circRNAs in the dataset. The average *F*_1_ score of circSLNN reaches 0.568, while the average *F*_1_ scores of iDeepE (Pan and Shen, 2018) and CRIP (Zhang et al., 2018) are 0.504 and 0.494, respectively. This suggests that the sequence labeling model can more accurately identify the position of the binding site, which is important for further verification of the interaction regions using biological experiments.

Despite the advantages over other methods, we can find that the overall accuracy is much lower than that computed on the short segments (the average *F*_1_ of 37 test sets is 0.790 as shown in **Table 1**). It is mainly due to the extremely imbalanced class distribution in this new test set. In training sets, the positive-to-negative ratio is 1:1, while when the full-length circRNAs are segmented, most of them contain no binding site at all. Although the model can handle imbalanced distribution to some extent as described in the *Investigation on Positive-to-Negative Data Ratio* section, the performance decreases greatly when the data set is severely imbalanced.

## Discussion

This study aims to develop a machine learning model for identifying RBP-binding sites on RNAs. The existing prediction methods consider this problem as a classification problem, which divide RNA sequences into fragments and predict whether or not binding sites exist in the fragments. To further predict the location and length of binding sites, we propose a sequence labeling model, circSLNN, which assigns a label to each base in fragments instead of the whole fragments, so as to provide more information of the binding regions. Besides, considering the lack of tools designed for circRNAs, circSLNN is specially trained by circRNA datasets. Although trained on circRNAs, circSLNN provides a general sequence labeling framework that can be applied to all types of RNAs.

Despite the enhancement of performance, this study is still a preliminary exploration on characterizing binding sites on circRNAs. The first limitation lies in the input features. As it is known that the interaction between RNAs and other molecules has complex mechanisms, especially the circRNAs that have not been well studies, the prediction of circSLNN is based only on circRNA sequences, which is a very limited information source. One future research direction is to incorporate more biological properties or domain knowledge related to circRNAs.

Second, although we have used a hybrid neural network, the proposed model structure is relatively simple. In recent years, not only new embedding training methods but also deep architecture have emerged in the field of natural language processing (Devlin et al., 2018), (Peters et al., 2018), which have achieved substantial improvement on a variety of tasks. Many of them could be adapted to biological sequence analysis, thus our network structure still has a lot of room for improvement.

Third, because the lengths of circular RNA sequences vary greatly, ranging from a few hundred to several millions, which seriously affects the training of the model. Most of the predictors including circSLNN are trained on short segments of RNAs, which may lose some information of whole RNAs and lead to high false-positive-rate. Better predictions based on full-length RNAs or longer segments are the focus of our future work.

## Conclusion

This study proposes a sequence labeling neural network for predicting RBP-binding sites on circRNAs, called circSLNN. To fully exploit sequence information, we train continuous embedding vectors for 10-mers of RNAs using the whole human genome sequences, and we construct a hybrid CNN–LSTM–CRF network to perform the sequence labeling task. The purpose of using a hybrid model is to combine the advantages of two deep architectures and to obtain better high-level abstract feature representations for classification. We train circSLNN on 37 datasets of circRNA fragments, and the average *F*_1_ score is 0.790. The experimental results show that it is feasible to use the sequence labeling method for identifying binding sites on circRNAs. Both the RNA fragment embedding vectors and the hybrid architecture contribute to improved performance. Compared with the classification model, it can more accurately label the position of the binding site on the full-length RNAs. The proposed model will help researchers study the circRNA–RBP-interactions and reveal regulatory functions of circRNAs.

## Data Availability Statement

All datasets generated/analyzed for this study are available at https://github.com/JuYuqi/circSLNN.

## Author Contributions

YJ, LY, YY and HZ designed the model. YJ and LY implemented the model and performed the experiments. YJ, LY, YY and HZ analyzed the results and drafted the article. YY and HZ supervised this work.

## Funding

This paper was partially supported by National Key Research and Development Program of China (No. 2017YFB0304100), Key Projects of National Natural Science Foundation of China (U1836222 and 61733011) and the National Natural Science Foundation of China (No. 61972251).

## Conflict of Interest

The authors declare that the research was conducted in the absence of any commercial or financial relationships that could be construed as a potential conflict of interest.
